# Supplementary figures and images for: Machine learning for automated electrical penetration graph analysis of aphid feeding behavior: Accelerating research on insect-plant interactions
Source: PLoS One. 2025 Apr 3;20(4):e0319484. doi: 10.1371/journal.pone.0319484 (PMC11967943; doi:10.1371/journal.pone.0319484)

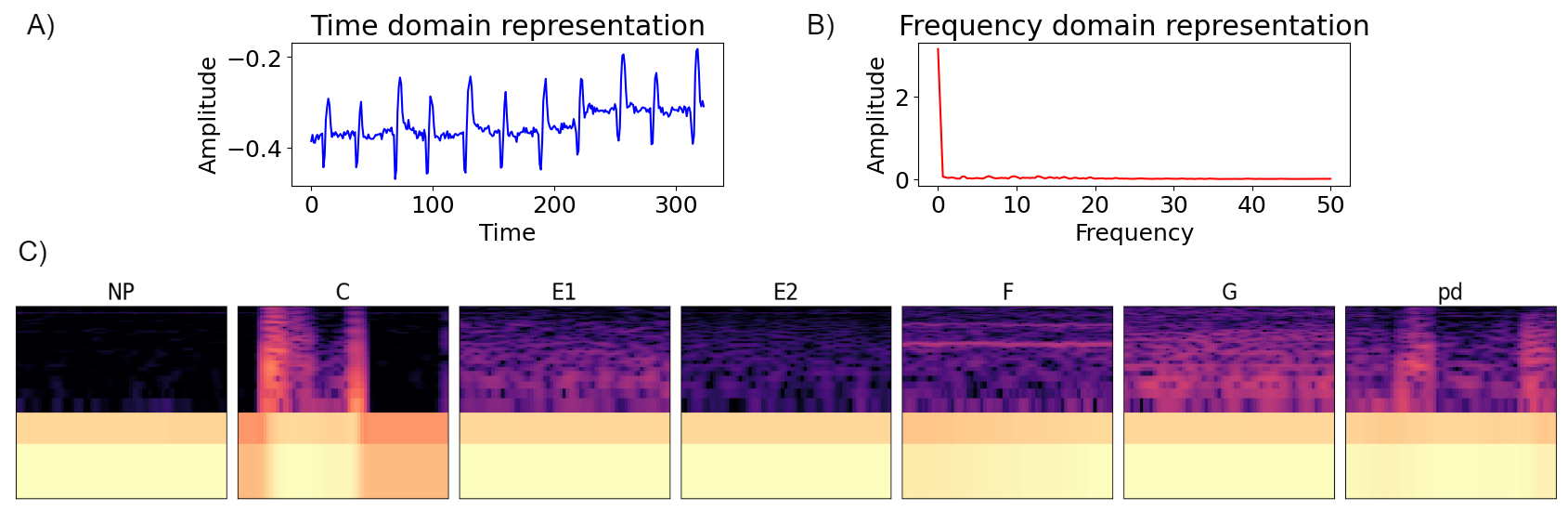

Supplement: S1 Fig — (TIF) [file pone.0319484.s001.tif]

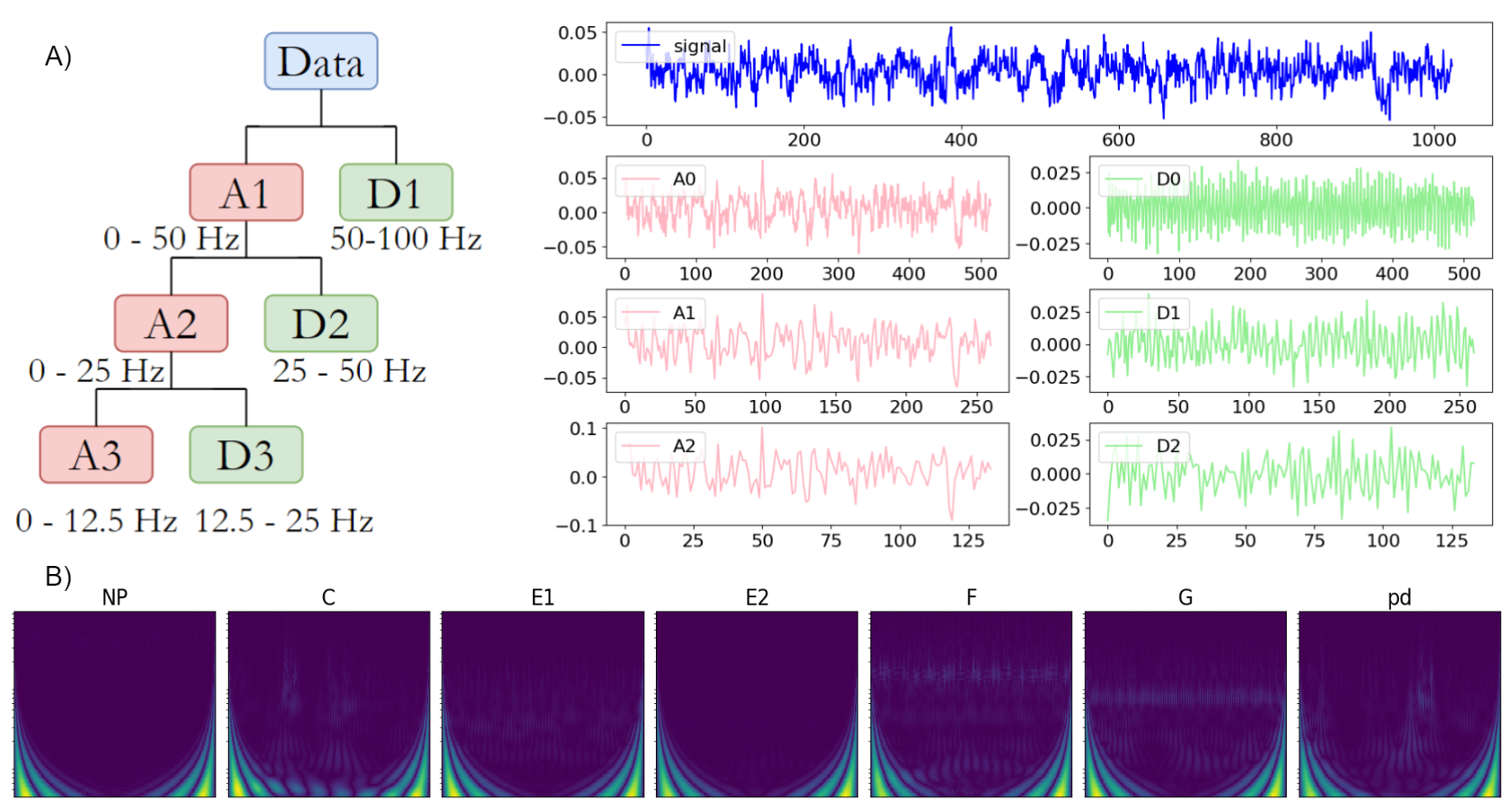

Supplement: S2 Fig — (TIF) [file pone.0319484.s002.tif]

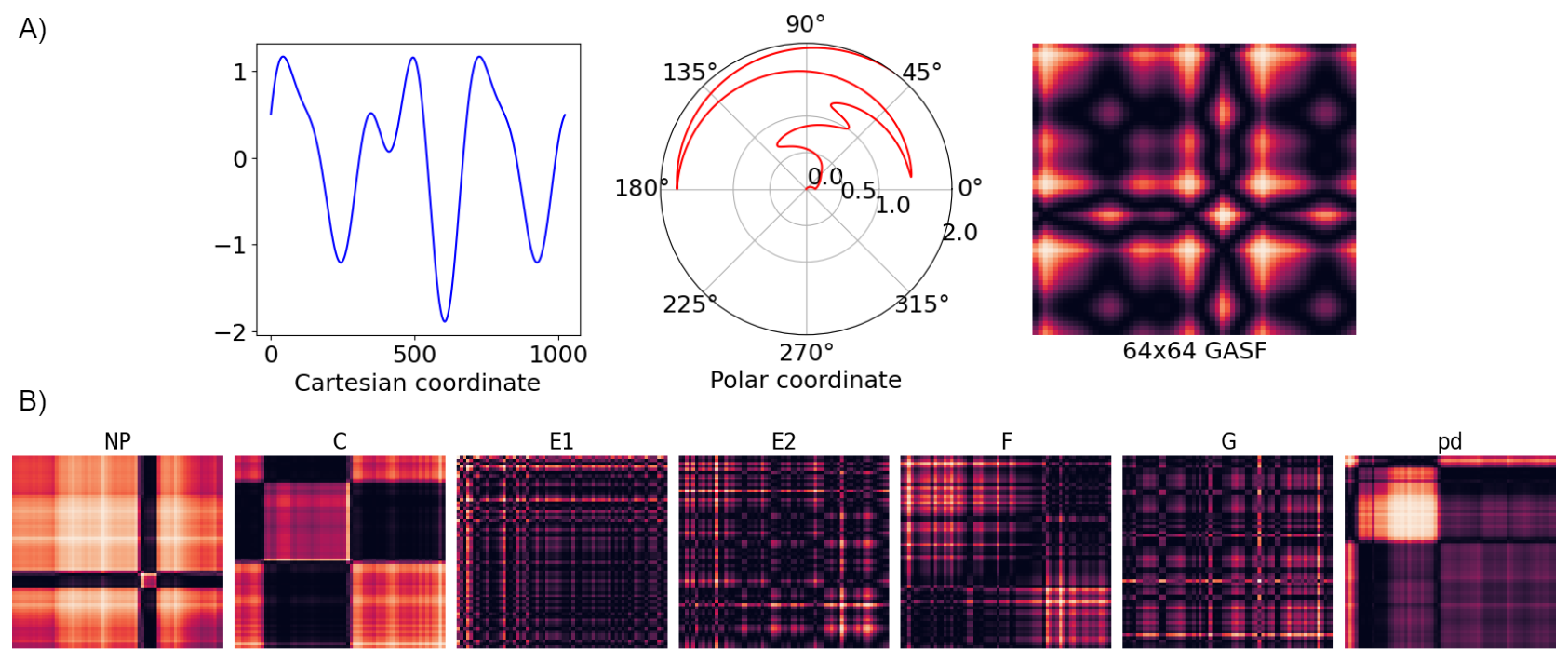

Supplement: S3 Fig — (TIF) [file pone.0319484.s003.tif]
