# Supplementary material for: Machine learning for automated electrical penetration graph analysis of aphid feeding behavior: Accelerating research on insect-plant interactions
Source: PLoS One. 2025 Apr 3;20(4):e0319484. doi: 10.1371/journal.pone.0319484 (PMC11967943; doi:10.1371/journal.pone.0319484)
Supplement: Attachment — Submitted filename: Dung_etal_Response to Reviewers.pdf [file pone.0319484.s004.pdf]

Response to Reviewer #1's comments

The paper titled "Machine Learning for Automated Electrical Penetration Graph Analysis of Aphid Feeding Behavior: Accelerating Research on Insect-Plant Interactions" presents a promising approach to enhance the study of insect-plant interactions using advanced computational techniques. The manuscript introduces a machine learning-based method aimed at automating the analysis of electrical penetration graphs (EPGs), which could significantly accelerate research in this field. However, several aspects of the manuscript need attention to enhance its scholarly contribution and readability.
Response: Thank you for your constructive comments and valuable suggestions, which helped us improve our manuscript. We have carefully revised it and addressed all your comments and questions.

The manuscript suffers from insufficient citations, particularly in sections where foundational techniques and historical context are discussed.
Response: We have edited the manuscript accordingly.

The abstract could be more concise. For instance, the following sentences can be combined to enhance clarity and brevity: "This automated approach promises to accelerate research in this field significantly and has the potential to be generalized to other insect species and experimental settings. Our findings underscore the value of applying advanced computational techniques to complex biological datasets, paving the way for a more comprehensive understanding of insect-plant interactions and their broader ecological implications."
Response: We thank the reviewer for this comment. The sentences have been edited as "This automated approach accelerates research in this field significantly and broadens insights into insect-plant interactions, showcasing the power of computational techniques for insect biological research."

Specific Comments:

o Historical methods can be cited appropriately: "Prior to EPG, real-time observation of aphid feeding was limited to histological methods, which only provide snapshots of the feeding process."
Response: We have removed this sentence from the manuscript and provided references that touch on some of the historical methods that were used (see Backus, 1994). (line 21)

o A citation is needed for the statement: "The initial EPG technique involved creating an electrical circuit by wiring the aphid and the plant, followed by recording the resulting voltage changes once the aphid closes the circuit by penetrating the plant tissue."
Response: This citation is found in the previous sentence. We have moved the citation to after the sentence in question to better reflect that the initial EPG technique was proposed in the Mclean and Kinsey, 1964 manuscript.

o The statement "Today, the EPG technique is the predominant method for studying hemipteran feeding behavior, largely supplanting earlier histological methods" should be supported by a systematic review or study.
Response: We have reworded this sentence to reflect that multiple methods are used to study feeding behavior previous to EPG and also included a review (Backus, 1994) of the history of the EPG technique, which discusses how EPG largely replaced these other methods.

o Avoid lump citations such as: "Neural Networks (CNNs) have shown great promise in insect behavior characterization [19–23]."

Response: We have noted the advice to avoid such lump citations. In this particular case we are trying to explain the diversity of the use cases for CNNs in studying insect behavior and providing users with some literature that documents these use cases. An adjustment has been made according to the reviewer's suggestion.

o Do not start a sentence with a parenthesis: "(Fig 2) describes the components of each dataset."
Response: We have updated this sentence accordingly. (line 112)

o In Fig. 1, consider adding pattern coding in addition to color-coding for different wave types shown in the graph.
Response: Thank you for your suggestion. We have explored several approaches to presenting the waveforms, and, after careful consideration, believe that the current format in this figure best conveys the intended information.

o Line 129, Page 7: Ensure to check and provide the missing reference for: "the 2nd leaf of seedlings at Zadoks stage Z1.2 (reference to be added)."
Response: We have added the missing reference.

o Table 1: Clarify what "n" stands for and provide an explanation of the variables.
Response: We have explained "n" as suggested.

o Number all equations for easy reference.
Response: We numbered all equations.

o Ensure proper citations in sections like "Gramian Angular Field" and "The complexity measures" paragraphs.
Response: We corrected the citations.

o Page 10, lines 209 to 213, can be omitted to maintain focus on the methods used and their results.
Response: We appreciate the reviewer's feedback and your efforts to help us refine the manuscript's focus. However, calculating these statistical features is a central component of our analysis. Including this explanation is essential for readers who may not have a strong computational background, as it ensures the accessibility and clarity of our methods. For this reason, we have decided to retain these sentences.

o The hyperparameter tuning method appears manual. Explain the rationale behind choosing ranges for each hyperparameter and discuss any alternative fine-tuning methods.
Response: We appreciate the reviewer's comment about the choice of range for tuning the hyperparameters. We believe that explaining every such choice is lengthy and unnecessary for most viewers from a biology background, who are the main targets of our work. Although the selection of initial parameter ranges is required for the ML models, our hyperparameter tuning process is done automatically. The model parameters were initialized by the default values given in the sklearn libraries. Having seen the performance, we search for a combination of parameters with a heuristic choice of ranges as long as the initial values are somewhere in the middle of the ranges. The same procedure was done for the DL algorithms. (line 389)

o Present the results of hyperparameter optimization clearly.
Response: We thank the reviewer for suggesting this. We have reworded the sentences and added more information to clarify our presentation.

o Avoid describing experimental setup in the results section: "For each ML model, we performed 3 groups of experiments with respect to 3 different techniques to preprocess its inputs, namely no transformation, Fourier transform, and wavelet transform (See Table 3). Each group contained experiments on 5 individual datasets having different settings. To assess the transferability of the ML models into different scenarios, we additionally conducted experiments with two representative models from the non-deep learning and deep learning groups."
Response: We have re-organized the Material and Methods section and the Result section so that the content is presented in a more systematic manner.

o Define terms like TP (True Positive), TN (True Negative), etc., in equations for clarity.
Response: We have included more explanation of the acronyms used in this section. (line 320)

o Discuss the importance of validation data and its results.
Response: To evaluate the ML algorithms, we carried out two evaluation tasks dedicated to assessing the performance of waveform segment classification and the overall annotation quality. We use 70% and 10% of the waveform sample database for the first evaluation task as the training and validation set, respectively. This 10% was used to guide our design for the algorithms. In other words, we adjust the models' hyperparameters (architecture-wise, such as the number of layers, number of neurons, normalization layers, or optimization-wise, such as learning rate, batch size, …) and judge their convergence based on this set.

o Report the percentage of samples for each waveform.
Response: Please refer to Table 2 for the exact numbers.

o Evaluate the accuracy of the models based on different waveforms.
Response: In our evaluation process, we perform both per-class (class-wise) and global evaluation by taking the mean of per-class metrics, and these validations are sufficient. Please refer to fig 5 and Fig 6 for details.

o Discuss the impact of varying sample numbers for different behaviors on the results.
Response: We admit that an imbalance in the number of samples (segments) of different waveforms/classes may affect ML model performance. However, this is unavoidable, given the nature of insect-feeding behavior. The patterns of aphid behavior expect some degree of variation according to biological factors such as the insect species and host plant species. For example, insects typically spend more time (for several hours) feeding after their mount-part penetrates leaf layers (e.g., the E2 waveform), making the waveforms in this behavior longer, leading to more waveform samples. We agree that an imbalance among the number of samples of 7 waveforms/classes makes it particularly harder to classify minority waveforms (such as pd). However, with rigorous data augmentation and model training, we achieved reasonable results for these minority waveforms in this study.

o Compare the results with those of other studies to provide a broader context.
Response: We have added a paragraph in the discussion section presenting the differences and advantages of our work in comparison to previous studies. (line 635)

o Ensure that results for "The complexity measures" are included and discussed.
Response: Thank you for the suggestion. However, selecting specific features that provide the best result for the traditional ML models is out of the scope of our work. For future work, it is a good idea to explore the performance of XGB with different features, as it is a light, quick and accurate model for annotating EPG waveforms.

Response to Reviewer #2's comments

The development of a machine learning approach to processing EPG data is a critical step in removing the barriers to wider application of this technology because (as the authors point out) this is a time-consuming task and prone to human errors. The author only used aphids but used aphids under a diverse set of conditions. That diversity is a great strength to this paper. It is also impressive that these models were small and did not require some massive supercomputer. I think both the successes and "failures" are interesting and have utility for developing the next step in whatever form that will take. One take-away message for me was that the segmentation approach worked well. Further refinements of the method are worth additional exploration.
Response: Thank you for your constructive comments and valuable suggestions, which helped us improve our manuscript. We have carefully revised it and addressed all your comments and questions.

Specific Comments:

Comment#1: At the end of the introduction, I am uncertain if the goal is to analyze only aphid waveforms or if the aphid data is being used as a demonstration of how this approach will work for all insects. Will the provided program work with any data, or is retraining necessary?
a. Retraining for every aphid species would limit usefulness of the program.
b. What happens if you train on three of the aphid species and then test on the fourth?
Response: The goal of this manuscript was to develop tools to automate the annotation of EPG data. While this manuscript only focused on EPG recordings from aphids, the same workflow would work on all EPG recordings obtained from the EPG Systems/Stylet+ systems. This research is a demonstration that applies to the suite of insects that have been recorded successfully on EPG Systems machines and have their waveforms defined. For non-aphid systems, the user would only need to modify annotation labels in the source code to match the convention for their insect.

The provided program will allow users to train their own models on their datasets. While most aphid waveforms appear similar, there can still be differences across experimental conditions or species. While retraining on each aphid species is more time-consuming, it allows for higher accuracy, given the variances in visual characteristics that can occur for each waveform. For this specific reason, we dedicated a set of experiments in which two representative models were trained and fine-tuned a large dataset formed by combining Bird cherry-Oat aphid 1 (BCOA1), Bird cherry-Oat aphid 2 (BCOA2), Cannabis aphid (CA), (Green Peach aphid) GPA and (Soybean aphid) SA.

Comment#2: It took reading the entire paper to be clear on Task 2. Somehow this should be clearer.
Response: We have reorganized the Materials and Methods section by presenting the EPG signal annotation and characterization before the Data section. This restructuring aims to clarify Task 2 earlier in the manuscript.

Comment#3: I cannot get Table 1 and Table 2 to agree. Table 1 states there were a total of 332 recordings. Each recording is 8 hours. With a segment of 10.24 seconds there are 2812.5 segments per aphid, and a total of 933750 possible segments (slightly fewer as I cannot work with a half segment). However, Table 2 has a combined total of 1142240 segments (total of train, validation, and test segments). Is this the effect of augmentation (Line 176)? Did augmentation have a different effect on different datasets?
a. In Table 2 the sum of the individual dataset for Train is less than the combined value.
Response: The main reason that your computation does not output the same number of waveform samples as ours is that we perform "padding" and "oversampling" on waveforms shorter than 10.24s, which we admit that we did not explain clearly. Specifically, for waveform samples (except pd) shorter than 10.24s, we pad the samples using the same method described in the Data Processing section. For

pd's samples, we also "oversampled", by reducing the window size (or step size) to 128 (allowing overlapping waveform samples). This strategy is particularly helpful when dealing with "pd" waveforms, which experienced a huge shortage of samples compared to dominant waveforms such as C and, E1, E2. To fix this, we have included a description of this data enrichment strategy in the manuscript.

Comment#4: What happens to segments that transition from one to another behavior? More what does that transition look like to the computer versus human. Do both choose the same break point?
Response: Currently, due to the sliding window approach used and the subtle inaccuracies that can arise due to flaws in initial training datasets, model overfitting, etc., there can be some slight differences between the breakpoints for a human and a computer. Note that, due to the sliding window approach, the breakpoints are often multiple of 10.24s (the window size). Hopefully, future updates will be able to address some of these inaccuracies.

Comment#5: There are some English language issues, both technical and in making the presentation clearer. I have caught some, but there are more. PLOS One does not edit, so I suggest trying something like the free version of Grammerly or a very careful rewrite. As is, the manuscript is readable. However, the mistakes will annoy some readers and likely cause some communication issues.
Response: We have edited the language to ensure that the manuscript is technically sound and with proper grammar.

Content issues:

Author abstract) You state four aphid species on four host plants. However, the methods have four aphid species on five host plants. The host plant issue is somewhat complicated in that not each aphid is given access to all hosts. I am not bothered by that, but it makes a simple statement less accurate.
Response: We have updated the text to reflect that this work was conducted on recordings obtained from 4 aphid species and 5 host plant species.

Line 11) Histology is never real-time. In histology you only know what the (now dead) insect was doing several hours (or days) in the past.
Response: We agree with the reviewer's point and have updated the text accordingly.

Line 19) Yes and no. The DC monitor was an improvement over the original AC monitors. However, with advances in electronics from 1964 to present we know that the AC monitor was also recording this information. However, in 1964 we had no way of uncovering this from the recorded signal. Forty years later, this is not a problem. AC or DC can be useful if one expands the use to past only-aphid because some insects are more sensitive to DC.
Response: We would like to mention that this line was only used to provide users a history of the EPG technique and its development, not to spark debate on AC vs DC EPG. Furthermore, we do not claim that AC EPG cannot record the emf component; we are simply providing the history that the invention of the DC EPG system, included in subsequent work by Tjallingi, was what enabled researchers to discover waveforms arising from both electrical origins, drastically increasing the usefulness of the technique. Furthermore, while there is some talk that certain insects are more sensitive to DC, this may be true, although there are very few studies to support these claims, and DC EPG has been used successfully in numerous hemiptera species. While there is a benefit to AC, DC, and even AC-DC EPG, our tool is powerful in that it will work on data obtained from each method so long as the user has digital-binary recording files with a sample rate of 100hz ending in a .D0X extension and an annotation file matching the columnar structure of the Stylet+ software with an extension of *.ANA.

Line 21) EPG does not supplant or replace histology. Histology is ESSENTIAL to interpret the waveforms for the first time. Once the correlation is established THEN EPG can be used to monitor Hemipteran feeding behavior. If a new waveform is discovered, then histology will again be critical in understanding what that waveform means biologically.
Response: We agree with your statement that histology is the vital groundwork that has enabled EPG to become so powerful; however, for groups with well-defined waveforms, such as aphids, it is certainly true that EPG has become a dominant method for studying feeding behavior. We have updated the text and removed our strong claim, and we agree that EPG cannot supplant or replace histology for specific studies. The sentence has been edited to read, "Today, the EPG technique stands as a leading method for studying hemipteran feeding behavior."

Line 26) The gold wire is not soldered to the nail. It is glued. The gold wire breaks too easily and therefore soldering is an inefficient attachment method. Neither Freddy nor Elaine teaches soldering, though soldering would work.
Response: We have updated the text to reflect that the gold wire is glued to a copper wire, which is then soldered to a brass nail.

Line 39) It can be shorter if the question is simple. More typically, 30 minutes grossly underestimates the time required. If my question is "Does the aphid ingest phloem" then I can scan all recordings for any presence of E2, and the task is quickly finished. If my question is "what are the differences" then I will record all behaviors, and the time required will greatly increase. Bad recordings, noise, recording time, and number of recorded behaviors all influence how long this process takes.
Response: You make an excellent point, and we have added further context on how bad recordings, noise, and ambiguous waveforms can influence annotation time.

Line 120) Are the aphid colonies from a single mother or are they a mixture of different clones?
Response: Our aphid colonies have typically been started with a few individuals (50 -100) collected from fields or greenhouses, which are then allowed to develop into larger colonies. From there, fresh plants are supplied to the numerous aphid clones and the colonies can be maintained for months to years. For the experimental aphids used in the data, numerous age-synchronization and normalization for individual mothers were used as the datasets were acquired over multiple years and in different labs.

Line 131) This is the training data. What steps did you take to check for the human errors in validated data? Same question for all datasets. I can partly answer the question in Tutorial_EPGDataset.ipynb in that it shows that some error checking was done.
Assuming that errors were corrected, how many errors did the humans make versus the ML program?
Response: The error checking included multiple EPG researchers browsing files from each of the datasets in the Stylet+ software, comparing the annotations and their locations on the waveforms. While we tried to error-check the training data, it is still possible that overlooked erroneous annotations are present in the data, as the task of reviewing so many waveforms is in itself very time-consuming. The text has been updated to remind the users that the model's effectiveness is only as good as the data it has been trained on, and that users should ensure that their annotations are as perfect as they can be.

Line 147) Was the biotype suitable to potato or might this be a laboratory rearing effect where the lab colony has largely lost any appetite for potato?
Response: The potential reasons why the aphid fed the way it did are discussed in the reference cited in this paragraph (Pitt et al., 2022). Cannabis aphids feeding on potatoes spent less time in E and more time in NP and F, suggesting that they performed/fed worse.

Line 176) In augmentation say (for discussion) I have a pd that lasts 4.2 seconds. I need to extend this to the left and right. How was that done? Say the 10.24 second segment has C-PD-C, I will then get three segments, the first C augmented right, the PD augmented left and right, and the second C augmented left?
Response: Assume that your pd was positioned at 10s-14.19s. From the midpoint of this sample (12.09s), extend the waveform to both sides to get a new sample at (12.09-10.24s) - (12.09+10.24s). Then, create (2048/128 + 1) overlapping segments of length 10.24s from this elongated pd sample. We accept the fact that a segment might contain a transition between two waveforms, which makes up for the fact that the sliding window approach would experience the same situation performing annotation on a complete recording. (line 247)

Line 258) What is dynamic time-warping distance?
Response: It is a metric used to measure the similarity between time series. However, we do not use this metric in our work.

Line 288) Consider defining TP, TN, FP, and FN. Yes, these abbreviations are common in the ML/AI literature but not all your readers will be familiar with that literature.
Someplace you might want to describe what happens with training, validation, and testing datasets. At least a couple of sentences.
Response: We have updated the Section so that the acronyms, as well as the evaluation protocol, are described more clearly. (line 320)

Line 291) More terms that are likely not familiar to EPG users even if many readers of PLOS One may know them.
Response: We have updated the Section so that the acronyms, as well as the evaluation protocol, are described more clearly.

Line 293) I do not know "w.r.t" and would there be a period after the t?
Response: "w.r.t." is the abbreviation for "with respect to". Yes, there should be a period after the "t". We corrected it to the complete term "with respect to" to avoid confusion.

Line 296) Please define "the second task" more clearly. I will guess: In the test dataset the goal is to look at how well the model predicts new observations. However, these observations are 10.24s segments, so the "second task" is to evaluate model performance for full recordings.
Response: In the Data Splitting subsection of the Data Processing section, we described the data preparation stage. Specifically, we use 90% of the recordings for training/validation/testing the model for each dataset. We also reserve 10% of the data (mentioned in Table 2, column Subset 2) for testing the automatic waveform annotation performance of the ML models on complete recordings. Recall that the idea behind our model is an annotation by first classifying the subsegments and then concatenating the classification results to get a complete annotation. Informally speaking, the model creates the big picture by combining its prediction on the little pieces. For training, we broke down one whole recording into segments and shuffled them before splitting them into training/validation/test sets, so it is more reasonable to assess the annotation process on a reserved set of recordings instead of immediately on "randomly shuffled" waveform samples. Finally, the term "Task 1" refers to the evaluation of the CLASSIFICATION performance on the little pieces. In contrast, the term "second task" (which we have changed to Task 2 to be more consistent) refers to the evaluation of the CONCATENATED classification results, which offers a view on the misalignment errors. (line 315)

Line 300) I am confused. I was picturing segments of 10.24s where there is no overlap between segments. This sounds like a moving window with the step size of the AD conversion interval (1/100 sec). In this case there would be 1024 segments containing each time step. Otherwise, there should be only one unless

augmentation results in overlap. Out of curiosity, in classifying the recordings in the second test, what is the comparison between where the human put the border between waveforms and the machine?
Response: In our work, we assigned ALL time steps, each with a label saying which feeding phase it belongs to. As the classification is made on 10.24s segments, each time step will take the label of the segment containing it. For example, the segment 0s-10.23s is classified as NP, then all the positions 0.00s, 0.01s, 0.02s, …, 10.23s are labeled as NP. Then, the next segment, 10.24s-20.47s, is classified as C, and all the positions 10.24s, 10.25s, 10.26s, …, 20.47s are labeled as C. In the end, we will have 2880000 labeled time steps for an 8h recording, from which we can tell the predicted position of each waveform. Then, the accuracy of the prediction is calculated by simply counting the number of time steps that have been predicted correctly. High accuracy means that more time steps were labeled correctly, and thus, the borders of waveforms are predicted more precisely. Please refer to the Workflow section (we reworded and added information to make the description more explicit) and (line 338) for details.

Line 361) reads like methods. Do you mean that all the data were used?
Response: We agree. We have moved the paragraph to the Method sections.

Table 6) What is the OR breakdown for the different datasets? This is more the average outcome, and the model trained on all the data may perform better or worse than individually trained models.
Response: The OR breakdown for different datasets was given in Fig 8 and Fig 9. Compared to the individually trained models, both XGB and 1DCNN experienced loss in the mean OR taken over 5 datasets from 84.5 to 80.6 and from 77.5 to 75.7, respectively (please see Table 4 and 5 for the OR of models trained on individual datasets). However, we note the following:

- The result was for the model using BOTH Grid Search (GS) and Feature Selection (FS). By additionally using Feature Selection, we accept the loss in accuracy in trade-off with the huge reduction in computational complexity as we calculate only 24 over 52 features. Most of the time required for training XGB is spent on the feature calculation process.
- By training with data from various backgrounds, we are essentially "distracting" the model, which generally results in a drop in accuracy. Nevertheless, the three metrics OA, avg-F1 and OR given in Table 6 of the model using only GS were only slightly lower compared to those recorded over of the individual datasets.

Line 515) These are not really experiments, not in the same sense as the aphid experiments.
Response: While we understand that this language is being used in two different contexts. We decided to keep the term 'experiments' as we are certainly experimenting with different hyperparameter tuning to achieve optimal training.

Line 519) Equating 256 with 2^8 should not be an automatic assumption for most biologists. They can work it out, but it is not an automatic response as it should be from a computer person.
Response: We have changed the exponential to numerical values.

Line 525) Behaviors should never overlap. There may be cases where behaviors overlap, say when feeding and oviposition are both recorded. There may be a few other cases where overlap is present, but these behaviors should never overlap.
Response: We agree that behaviors can never overlap, they are discrete phenomena. We have changed the language to reflect that pd is nested within C, not that C and pd overlap.

Figure 10) Can you make another line that is white when there is agreement and black where there is disagreement? One thing I am interested in is where disagreement takes place. Is this mostly at the transition zones, or entire behavioral events? Figure 10 suggests at least sometimes it is the entire events.

Response: In fact, the function for plotting comparison between the prediction and ground-truth annotation of our package has an argument "range" which allows users to display the comparison at a specific part of the recording (say 1000s-1100s). We have updated the description of the function accordingly.

Figure 11) Very interesting. There were more errors when a recording was not set up and the signal maxed out. It is difficult to read at this scale, but after G there are two NP spikes and then a C spike. It looks like the C spike is a ground-truth error, but I might disagree with this assessment if I could see more detail. I used the clear C at the beginning of the recording to make this assessment. Obviously, the model made some serious mistakes.
Response: For the C spike in question (Figure 11, ~18000 s), this does seem to be a ground truth error after double checking the recording in question. Directly after G ends (which we now believe is F and was mislabeled for G; G typically will not directly end into NP, it needs to come back out into C or F for that to make sense) the two C spikes in question are genuine. The spikes after the second large C spike (~ 7.5V amplitude) are all NP and the next genuine C does not start until the next large spike that reaches around a 7.5V amplitude. Interestingly, the model was able to correctly predict these waveforms, likely due to the recordings used for training being more accurate than this recording in question. This highlights how these models can be more accurate than human labels given that they were trained on quality ground truth annotations. For recordings with data that goes beyond the bounds of recording for the EPG devices, either removing the files in question or possibly adding a new label such as over-voltage ("OV") or under-voltage ("UV") to the ANA files before training could be used to be able to detect such over and undervoltage events. It is crucial for the initial user to adjust the gain appropriately during the recording duration to ensure quality recording. In case you would like to verify, we are happy to share the data of this recording, and please refer to our package for making detailed visualization.

Line 528) What do you mean by "irregular samples?"
Response: We meant the waveform samples whose behavior are not consistent with the general observed behavior (for example, E2 samples which do not display the characteristic periodicity to E2)

Line 553) Are you saying that I could just use the 1DCNN model and analyze my melon aphid data? Or do you mean that I could use the 1DCNN approach and train my own model where initially I would need a large data set to do the training?
Response: Ideally, the user would create/train their own model for their species of interest (assuming they have a medium-large collection of recordings i.e. 30-100). For the moment, we have only provided a species-wide model that can be used for initial model training and inference. The user can either use our model for automatic annotation of aphids or train their own model with their own annotated data (which is always the ideal case). In the future, we will add functionality allowing the user to perform "transfer learning", meaning that our model can then be further trained with data from your species of interest.

Line 556) So you think the issue is short duration rather than the number of transitions? Each PD generates two transitions C-PD and PD-C.
Response: It is possible that there are a few reasons for the issue in question. Other potential causes could also be the correlation of C and pd (i.e. pd being nested within C) and the two transitions that you mention.

Line 556) detailed
Response: We corrected it.

Line 557) Do not start a sentence with "(Fig 10)"
Response: We adjusted it accordingly.

Line 558) matches
Response: We corrected it.

Line 560) waveforms
Response: We corrected it.

Line 586) Not so minor. I run an experiment and there are 50,000 events over the entire experiment. My error rate of 10% results in (roughly) 5000 mistakes. Depending on the nature of the mistakes it could be worth doing everything manually versus trying to find and correct that many errors. This is still a task well worth the effort, but 90% accuracy is not quite good enough to be transformative. At best, slightly useful if you are careful.
For PD, is prediction accuracy a function of PD length?
Response: We agree with the sentiment that errors can become untenable when the datasets get larger and larger and that the users need to be careful in blindly accepting the predicted annotations and provide adjustments when needed. In terms of checking whether it is easier to predict PDs if they are longer or shorter, we have not done that analysis but will look into this in the future. The prediction accuracy of PD is the same as those of the other waveforms.

Line 585) Difficult to tell due to scale. Without exception there will be an E1 before every E2. I am having trouble distinguishing the brown of NP and the olive of PD. However, it looks like the 2D-CNN model has a NP-E2 transition at 6000 seconds.
Response: We agree that the visualization might not be sufficient to display our presented result. The issue you mentioned is precisely correct, and it is also one of the disadvantages of our model. Our model cannot consider the relationship between waveforms (such as E1 must precede E2, or pd must occur inside C). Thus, future developments in terms of prediction algorithms could greatly improve our results.

Line 628) You are making this up. However, the overall premise is true: EPG is a labor-intensive time-consuming task prone to human error and ML is a viable solution. I think this paper shows that more conclusively than similar papers.
Response: We appreciate your belief that our manuscript more convincingly demonstrates the viability of machine-learning techniques for these tasks. However, the assertion that EPG is time-consuming, labor-intensive, and prone to operator error is not unfounded.

Line 11) The citation is hanging as a sentence all its own.
Response: We fixed it.

Line 13) Needs editing. Trying to combine history with method and method development. Just simplify these few sentences.
Response: We have edited these sentences.

Line 16) Be clearer. Enhancing EPG equipment (AC, DC, AC-DC) is different than understanding or interpreting the biological meanings of the waveforms. That is also different from improving methods like selecting wire thickness, metal (gold versus platinum) and type of adhesives used to attach the wire to the insect. Histology does not influence the electronic development of the equipment.
Response: We have updated the text accordingly.

Line 28) Is the electrode or the soil connected to EPG circuit?
Response: We have updated the text accordingly to reflect that the probe inserted into the soil connects to the EPG recording device.

Line 33) In manually annotating the file the user has defined the waveforms and durations. The various feeding behaviors are not manually calculated.
Response: After annotation, the user has to organize their data directories, then manually open up their favorite EPG parameter calculation software, select the recordings they would like to calculate, fill out metadata about the recordings, etc. (if you are at all familiar with the Sarria Excel workbook (Sarria et al., 2009) you understand just how manual that process was).

Line 34) The Excel workbooks calculate variables from the sequence of observed behaviors.
Response: We have edited the text to say that the workbooks calculate various parameters associated with feeding behaviors.

Line 129) Need to replace "reference to be added". Is this "in press" or "not submitted?" Make this a valid acknowledgment of where the data came from, and then change it (if possible) as you revise the manuscript.
Response: This was a review oversight, and we have added the citation!

Line 129) datasets (you have more than one).
Response: We have adjusted this line accordingly

Line 134) In the entire manuscript remove spaces before periods at the end of sentences. It is a good idea to also use global replace to remove any double spaces generated as part of editing the document (if present).
Response: We edited the manuscript accordingly.

Line 179) is "Task" here "Subset" in Table 2?
Response: For Task 1 and Task 2, we used Subset 1 and Subset 2, as described in the Data Splitting subsection.

Line 187) Delete powerful.
Response: We have deleted it.

Line 203) "converts a" and generally corrects the English.
Response: We corrected it.

Line 257) What is TSF? This is the only use of this acronym.
Response: TSF stands for Time-Series Random Forest. We rephrased our idea without using the acronym as this is its only use.

Line 259) You say three and list four.
Response: We corrected it.

Line 260) What are LGBM, GBM, and CatBoost?
Response: The acronyms stand for Light Gradient Boosting Machine, Gradient Boosting Machine, and Category Boosting, which are the algorithms that work based on the gradient boosting architecture. These are well-known in the AI/ML community.

Line 261) showed
Response: Corrected

Line 262) So not all state-of-the-art performances are equal?
Response: The performances of ML algorithms may vary according to various factors (e.g., the type of data, the choice of hyperparameters, the characteristics of datasets). Despite this, one may choose state-of-the-art for their application as they work better than others.

Figure 7) Left graph axes are difficult to read.
Response: We could not make it more readable since there were too many lines.

Line 418) What are colos?
Response: It was colors. We have corrected it.
